# Intravenous vitamin C in the treatment of post-laser hyperpigmentation for melasma: A short report

**DOI:** 10.1080/14764170802187193

**Published:** 2008-12-17

**Authors:** Georgia Siow Kiang Lee

**Affiliations:** TLC Medical Practice, Singapore

**Keywords:** Intravenous, melasma, post-laser hyperpigmentation, vitamin C

## Abstract

Melasma is difficult to treat. Vitamin C, topical and by iontophoresis, has been shown to be useful. When lasers are used, there is a significant incidence of post-laser hyperpigmentation. There is no single established treatment for the latter. The case history of a 51-year-old Chinese woman is presented. Intravenous vitamin C appears to be useful in treating this complication.

## Introduction

Melasma is often difficult to treat. It is more common in females and is associated with sun exposure, pregnancy and contraceptive pill use. When severe, it can be socially embarrassing for patients. Treatment involves broad-spectrum (UVA+UVB) sunscreen and topical hydroquinone. Other lightening agents include retinoic acid (tretinoin) and azelaic acid. Combination therapies such as hydroquinone, tretinoin, and corticosteroids are thought to be superior to monotherapy. Lasers are used in cases of refractory melasma, but the incidence of post-inflammatory hyperpigmentation is high (1), and more so in Asian populations with higher epidermal melanin content (2). When it occurs, treatment with hydroquinone cream with other depigmentating agents speeds its resolution (3), which can take several months. Intravenous vitamin use to treat this distressing complication is described.

## Case report

**Figure 1:**
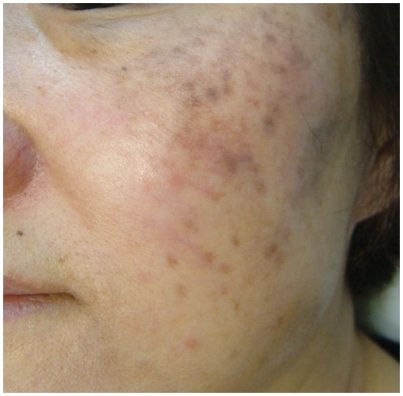
Before treatment with iontophoresis vitamin C.

**Figure 2:**
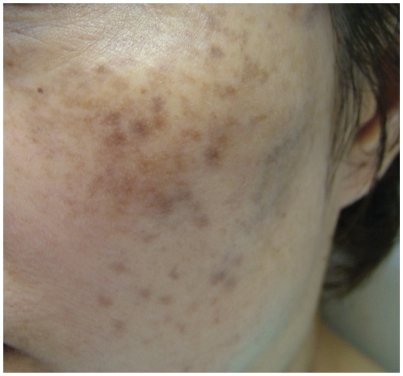
After seven sessions with iontophoresis vitamin C.

**Figure 3:**
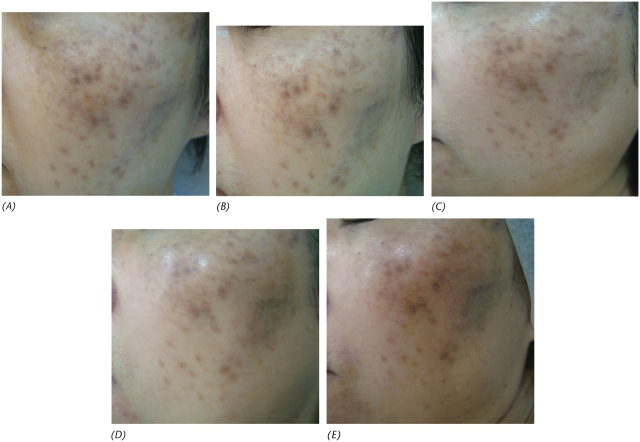
After the first (A), second (B), sixth (C), 10th (D) and 11th (E) laser sessions with iontophoresis vitamin C.

A 51-year-old postmenopausal Chinese woman presented with significant melasma of many years duration with no response from sunblock, chemical peeling and depigmentation cream obtained from another practitioner (Figure 1). Depigmentation cream (retinoic acid 0.025%, hydroquinoine 5% and triaminolone 0.1%) was applied to the affected area at night. Concurrent vitamin C iontophoresis was started with some improvement after seven sessions. However, she developed excessive dryness from the treatment (Figure 2). A course of monthly Q-switched 1064 nm Nd:YAG laser treatment with settings at spot size 7 mm and 2.1 J/cm^2^ was added to the vitamin C iontophoresis regime with some improvement in the skin condition initially (Figure 3).

She developed post-laser hyperpigmentation after 11 laser sessions and intravenous vitamin C (7 g) was added after 18 months of therapy. A total of three doses of intravenous vitamin C, 7 g each time, were administered; the first two doses were given a week apart, 1 month after the laser treatment which caused her significant hyperpigmentation. She returned for follow-up after a 5-month lapse due to work commitments with no other treatments prescribed in the interim, when a 12th session of laser treatment was performed together with a third concurrent dose of intravenous vitamin C (7g).

**Figure 4:**
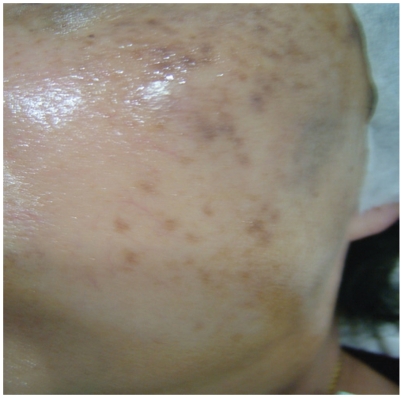
After the 12th laser session with three sessions of intravenous vitamin C.

She had significant improvement in skin pigmentation after this last session which is lasting without any post-laser hyperpigmentation noted (Figure 4).

## Discussion

Post-laser hyperpigmentation is a significant complication and requires considerable preoperative counseling and effort in management (4). Patients with darker skin tones are at a higher inherent risk of side effects from laser surgery. Although advances in laser technology and individualized treatment parameters have reduced the incidence of undesirable postoperative sequelae, these risks can never be completely eliminated. Hence, thorough preoperative preparation and education regarding the risks of cutaneous laser therapy is essential (5). The Q-switched (Qs) Nd:YAG laser is specially indicated in targeting melanin for the treatment of melasma, although it is associated with a significant incidence of post-inflammatory pigmentation. This is especially distressing to the patient if the indication for laser therapy is localized hyperpigmentation in the first place.

There is considerable literature on the topic of intravenous vitamin C, a strong anti-oxidant, which plays an important role in maintaining physiological states. In dermatology, vitamin C is used in the treatment of various skin problems such as depigmentation of hyperpigmented spots (6). Melanogenesis is caused by enzymatic conversion of tyrosine to melanin pigments. As ascorbic acid has the ability to inhibit peroxidase and thus melanin synthesis, the University of Taiwan has proposed investigating the use of intravenous vitamin C in improving skin hyperpigmentation in chronic hemo-dialysis patients. However, vitamin C has limited stability and permeability (7). Improving vitamin C delivery by iontophoresis appears useful (8). Padayatty et al. studied vitamin C pharmacokinetics, comparing oral and intravenous routes in 17 healthy hospitalized volunteers. They concluded that only intravenous vitamin C produces high plasma concentrations (9). Hence, it is logical to postulate that intravenous vitamin C may be effective in the treatment of post-laser hyperpigmentation.

The use of high-dose vitamin C in curing a variety of conditions such as the common cold to cancer is quite controversial. However, the debate remains such that the FDA is currently sponsoring a clinical trial of intravenous vitamin C in cancer. There is little evidence that ascorbate, as all naturally occurring substances, has any harmful effects, except in individuals with a rare genetic predisposition. A theoretical side effect could be on oxalate metabolism, which predisposes to kidney stones (10). Animal experiments indicate there is little risk of overdosage in the regimen used clinically. There is also a tendency towards hypoglycemia during intravenous administration.

## Conclusion

This case illustrates the typical problems associated with treating melasma. This includes prolonged courses of therapy using multiple modalities. Vitamin C is useful as evidenced by the response to iontophoresis. But with the added complication of post-laser hyperpigmentation, it appears that intravenous vitamin C in a moderate dose did offer a significant benefit in this patient. Further experience is required with vitamin C when administered by the intravenous route.
